# The Transcription Factor *ZmMYBR24* Gene Is Involved in a Variety of Abiotic Stresses in Maize (*Zea mays* L.)

**DOI:** 10.3390/plants14132054

**Published:** 2025-07-04

**Authors:** Liangliang Bao, Wen Sun, Jiaxin Wang, Yuyang Zhou, Jiahao Wang, Qi Wang, Dequan Sun, Hong Lin, Jinsheng Fan, Yu Zhou, Lin Zhang, Zhenhua Wang, Chunxiang Li, Hong Di

**Affiliations:** 1 College of Agriculture, Northeast Agricultural University/Key Laboratory of Germplasm Enhancement, Physiology and Ecology of Food Crops in Cold Region, Ministry of Education/Engineering Technology Research Center of Maize Germplasm Resources Innovation on Cold Land of Heilongjiang Province, Harbin 150030, China; 15794892115@163.com (L.B.); 15245336855@163.com (W.S.); 15103869531@163.com (J.W.); zyyneau@163.com (Y.Z.); 17332328082@163.com (J.W.); wwangqi097@163.com (Q.W.); zhouyu0924@126.com (Y.Z.); neauzla@163.com (L.Z.); zhenhuawang@neau.edu.cn (Z.W.); 2 Institute of Forage and Grassland Sciences, Heilongjiang Academy of Agricultural Sciences, Harbin 150086, China; sundequan0451@163.com (D.S.); linhongltt@163.com (H.L.); fanjinsheng0424@163.com (J.F.)

**Keywords:** maize, *ZmMYBR24* gene, adversity stress, functional validation, RNA-seq

## Abstract

MYB transcription factors constitute a diverse and functionally versatile family, playing central roles in regulating plant responses to a range of abiotic stressors. Based on previous research, we identified and characterized a maize MYB transcription factor gene, *ZmMYBR24*, which is involved in responses to salt, alkali, and low-temperature stress. This study aimed to investigate the function and mechanism of *ZmMYBR24* in response to salt, alkali, and low-temperature stresses. We hypothesized that *ZmMYBR24* regulates biosynthetic pathways to influence maize resistance to multiple abiotic stresses. The results indicate that *ZmMYBR24* expression was markedly upregulated (*p* < 0.01) and the fold-change in gene expression ranged from 1.54 to 25.69 when plants were exposed to these combined stresses. Phenotypically, the *zmmybr24* mutant line exhibited more pronounced inhibition of seedling and root growth under stress compared to the wild-type B73 line. Based on a correlation expression pattern analysis and mutant line evaluation, *ZmMYBR24* was confirmed to be a positive regulatory transcription factor for multiple types of abiotic stress resistance. An RNA-seq analysis of both lines revealed differentially expressed genes (DEGs), with gene ontology (GO) and KEGG enrichment analyses indicating that *ZmMYBR24* may mediate stress responses by modulating the expression of genes involved in flavonoid biosynthesis. Notable differences were observed in the expression of pathway-associated genes between the mutant and wild-type plants. A haplotype analysis across 80 inbred maize lines revealed 16 *ZmMYBR24* coding region haplotypes—comprising 25 SNPs and 17 InDels—with HAP12 emerging as a superior haplotype. These results demonstrate that *ZmMYBR24* enhances maize yields by regulating the flavonoid biosynthesis pathway in response to adverse climatic conditions including salt, alkaline conditions, and low temperatures. Collectively, these findings offer novel insights into the molecular mechanisms underlying maize adaptation to combined abiotic stresses and lay the groundwork for breeding programs targeting multi-stress resistance.

## 1. Introduction

Salt stress, which is primarily caused by neutral salts such as NaCl and Na_2_SO_4_ [1,2], significantly reduces seed water absorption and germination rates [3], thereby inhibiting plant growth and development. Alkali stress, induced mainly by alkaline salts such as NaHCO_3_ and Na_2_CO_3_ [1,2], increases exchangeable sodium levels, disrupting plant physiological and metabolic functions. These disruptions can lead to severe growth inhibition or even plant death [4,5]. Under combined salt–alkali conditions, seed germination, growth, development, and biomass accumulation are more severely compromised [6,7]. Similarly, low-temperature stress substantially impairs crop growth and yields [8]. In Northeast China, chilling events now occur approximately every 3.5 years, causing considerable agricultural losses. When salt and alkali stress co-occur with occasional low-temperature events, the resulting composite stress poses even greater challenges to crop productivity. As such, elucidating the molecular mechanisms underlying plant responses to these combined stresses and identifying key regulatory genes have become research priorities.

Advancements in stress-related gene discovery have yielded promising results. In rice, *STG5* enhances salt tolerance by transcriptionally regulating *OsHKT* family members, maintaining Na^+^/K^+^ homeostasis [9]. Loss of *RST1* function promotes *OsAS1* expression, improving nitrogen use efficiency via enhanced asparagine synthesis and limiting NH_4_^+^ accumulation, ultimately mitigating yield losses under salinity stress [10]. Meanwhile, *ATT1/SD1* and *ATT2/GNP1* regulate gibberellin biosynthesis, and their function suppresses the ROS scavenging system, making plants more susceptible to alkali–heat stress due to excessive ROS buildup [11]. *CTB5* interacts with *OsHox12* to coordinate anther development and enhance seedling-stage cold tolerance through GA metabolism and ABA signaling modulation [12]. However, these genes largely confer resistance to specific, individual stressors. Genes that offer broad-spectrum resistance to salt, alkali, and low-temperature stresses remain rare and underexplored.

Maize (*Zea mays* L.) is a globally important cereal crop used for food, feed, and industrial applications. As stress-resilient germplasm resources expand, numerous maize genes conferring abiotic stress resistance have been identified. For instance, *ZmESBL* and its homologs modulate Casparian strip lignification to control Na^+^ loading in the stele, enhancing salt exclusion and tolerance [13]. *ZmNAC84* overexpression improves salt tolerance by upregulating *ZmCAT1*, boosting catalase activity and reducing H_2_O_2_ accumulation [14]. The mitochondrial sterility gene *ORF355* enhances salt tolerance by adjusting cellular metabolism [15]. Furthermore, the atypical G-protein γ subunit encoded by *SbAT1* suppresses aquaporin phosphorylation, regulating H_2_O_2_ efflux and improving alkali tolerance, which is further elevated by *AT1/GS3* knockout [16]. A genome-wide association study involving 205 inbred maize lines identified *COOL1*, a transcription factor on chromosome 3, as a negative regulator of cold-responsive genes and a modulator of low-temperature tolerance [17]. However, all of these genes address single stress factors, underscoring the need for genes that confer multi-stress resilience.

MYB transcription factors are one of the largest TF families in plants and play integral roles in mediating responses to abiotic stress. Rice genes such as *OsMYB48-1*, *OsMYB2*, *OsMYB6*, and *OsMYB3R-2* have also been implicated in both salt and cold stress responses [18,19,20,21]. In maize, *ZmMYB3R* enhances salt tolerance by activating ABA-responsive genes and antioxidant enzymes [22], while *ZmMYB30* broadly regulates adversity-responsive genes to improve stress resilience [23]. *ZmMYB-IF35* boosts cold tolerance via the activation of stress-related genes, including *AtCBF2*, *AtCBF3*, *AtCOR1*, and *AtCOR2* [24]. *ZmMYBR24* is a newly identified gene from the MYB transcription factor family. It belongs to the same family as the aforementioned genes; it represents a distinct gene with a conserved domain. Based on transcriptome data, *ZmMYBR24* may participate in the response to adversity stress by regulating certain biosynthetic pathways.

Single-nucleotide polymorphism (SNP) refers to DNA sequence diversity caused by variations in single nucleotides at the genomic level [25]. As the most abundant form of DNA sequence variation with high frequency, SNPs serve as an important molecular basis for species genetic diversity. Researchers have investigated the genetic diversity of Ethiopian sorghum using SNP markers, genotyping 359 individuals representing 24 landraces with 3001 SNP markers [26]. Through SNP marker-assisted selection, the salt-tolerant gene hst1 from ‘Kaijin’ was precisely introgressed into the high-yielding rice variety ‘Yukinko-mai’. Combined with biotron acceleration technology, the salt-tolerant germplasm ‘YNU31-2-4’ was developed within 17 months (six generations), exhibiting 93.5% genomic similarity to the wild type while demonstrating significant salt tolerance and yield advantages under salt stress during both the seedling and reproductive stages [27].

Our previous transcriptomic analyses under salt, alkaline [28], and low-temperature [29] stress conditions identified *ZmMYBR24* as a differentially expressed gene exhibiting consistent upregulation under all three conditions. In this study, we characterize *ZmMYBR24* and demonstrate its involvement in co-regulating maize responses to salt, alkali, and low-temperature stress. Based on a bioinformatics analysis, yeast autoactivation verification, gene expression pattern analysis, mutant functional validation, RNA-seq, and sequence variation analysis, we aim to achieve the following objectives: (1) determine the basic characteristics of the *ZmMYBR24* gene; (2) investigate the expression patterns of *ZmMYBR24* in the wild-type inbred line B73 and the mutant line *zmmybr24*; (3) assess the stress resistance capacity of wild-type and mutant lines under salt, alkali, and low-temperature stress; (4) identify differentially expressed genes and key pathways under salt, alkali, and low-temperature stress; (5) screen superior haplotypes and stress-resistant inbred maize lines for subsequent breeding; (6) conduct a preliminary exploration of the response mechanism of *ZmMYBR24* to multiple abiotic stresses during breeding.

## 2. Results

### 2.1. Basic Characterization of ZmMYBR24

**Figure 1:**
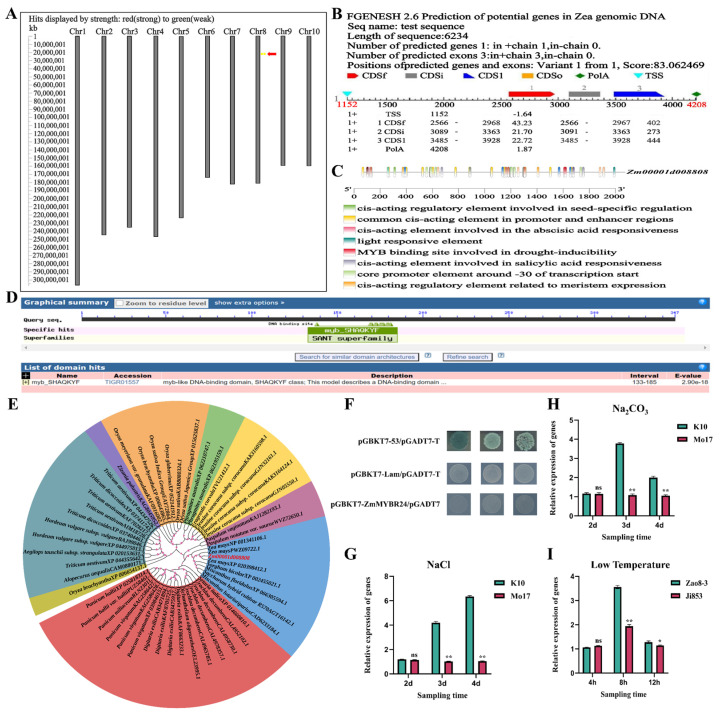
Basic characteristics of *ZmMYBR24*. (**A**) Chromosomal localization of *ZmMYBR24*. (**B**) Gene structure of *ZmMYBR24*. (**C**) Flanking sequence of *ZmMYBR24*. (**D**) Conserved protein domains of *ZmMYBR24*. (**E**) Phylogenetic tree of ZmMYBR24 protein. (**F**) Autoactivation verification of *ZmMYBR24*. (**G**) Relative expression levels of *ZmMYBR24* under salt stress. (**H**) Relative expression of *ZmMYBR24* under alkali stress. (**I**) Relative expression of *ZmMYBR24* under low-temperature stress. Data are presented as the mean ± SD (*n* = 3 independent biological replicates). Statistical significance (*p* < 0.05) was determined by one-way ANOVA with multiple comparisons. ns indicates no significant difference, * and ** indicate significant differences at *p* < 0.05 and *p* < 0.01 levels, respectively.

The *ZmMYBR24* gene is located in bin 8.03 of chromosome 8 in maize (Figure 1A) and spans 2234 bp, comprising three exons. It harbors a complete open reading frame of 1044 bp, encoding a basic, hydrophobic, and unstable alkaline protein (Figure 1B; Table S1). Promoter analysis revealed a stress-responsive regulatory region, containing core motifs such as the CAAT-box and TATA-box, alongside multiple abiotic-stress-responsive elements, including MBS, ABRE, and ABRE3a (Figure 1C).

The protein’s conserved domain structure includes a myb_SHAQKYF motif spanning amino acids 133 to 185, placing it within the structurally conserved SANT superfamily (Figure 1D). ZmMYBR24 lacks transmembrane domains and signal peptides, suggesting an intracellular localization and non-secretory nature. It contains one ZF-CCHC and one HTH-MYB domain, classifying it as a 1R-MYB/MYB-related transcription factor (Figure S1). The phylogenetic analysis showed high similarity to two maize proteins: LOC103634904 (XP_020398412.1) and LOC110973014 (NP_001341106.1) (Figure 1E). All aligned protein sequences, including the Zm00001d008808 transcription factor-like protein, are listed in Table S1.

A yeast two-hybrid (Y2H) assay revealed that the co-transformation of pGBKT7-*ZmMYBR24* and pGADT7 failed to support growth on SD-Trp-Leu-His-Ade/X-α-gal/AbA (0 ng/mL) medium, indicating that *ZmMYBR24* lacks transcriptional self-activation activty (Figure 1F).

An expression analysis under stress conditions demonstrated the dynamic regulation of *ZmMYBR24*. Under salt stress, expression peaked at day four (Figure 1G), while, under alkali stress, expression reached a maximum at day three before declining (Figure 1H). Notably, inbred line K10, which shows greater salt and alkali tolerance, exhibited significantly higher *ZmMYBR24* expression than the sensitive line Mo17. In response to low-temperature stress, expression peaked at 8 h and subsequently declined. Expression levels were markedly higher in the cold-tolerant inbred line Zao8-3 compared to the cold-sensitive line Ji853 (Figure 1I).

### 2.2. Functional Characterization of ZmMYBR24 in Stress Resistance

**Figure 2:**
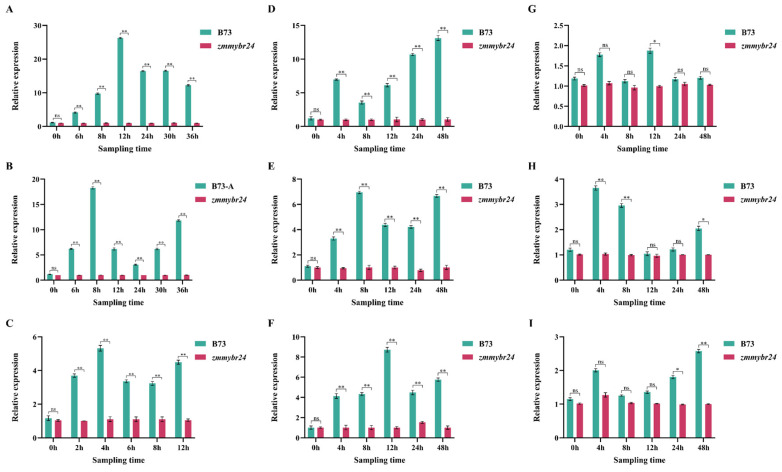
Functional characterization of resistance during the germination and seeding stages. (**A**) Relative expression of the *ZmMYBR24* gene in seed embryos under NaCl stress. (**B**) Relative expression of the *ZmMYBR24* gene in seed embryos under Na_2_CO_3_ stress. (**C**) Relative expression of the *ZmMYBR24* gene in seed embryos under low temperature stress. (**D**) Relative expression of the *ZmMYBR24* gene in leaves under NaCl stress in the seedling stage. (**E**) Relative expression of the *ZmMYBR24* gene in leaves under Na_2_CO_3_ stress in the seedling stage. (**F**) Relative expression of the *ZmMYBR24* gene in leaves under low temperature stress during the seedling stage. (**G**) Relative expression of the *ZmMYBR24* gene in roots and leaves under NaCl stress in the seedling stage. (**H**) Relative expression of the *ZmMYBR24* gene in roots and leaves under Na_2_CO_3_ stress in the seedling stage. (**I**) Relative expression of the *ZmMYBR24* gene in roots and leaves under low temperature stress in the seedling stage. Data are presented as mean ± SD (*n* = *3* independent biological replicates). Statistical significance (*p* < 0.05) was determined by one-way ANOVA with multiple comparisons. ns indicates no significant difference, * and ** indicate significant differences at *p* < 0.05 and *p* < 0.01 levels, respectively.

A homozygous *zmmybr24* mutant was generated via a point mutation (A→C) at position 973 (Figure 2A). Under NaCl stress, *ZmMYBR24* expression in the embryo peaked at a specific time point, decreased, and then rose again (Figure 2A). A similar pattern was observed under Na_2_CO_3_ stress (Figure 2B) and low-temperature stress (Figure 2C). Across all three stress conditions, *ZmMYBR24* expression was significantly higher in the wild-type B73 line than in the *zmmybr24* mutant.

During seedling development under NaCl stress, *ZmMYBR24* expression in leaves peaked at 4 h, fluctuated, and reached a secondary peak at 48 h (Figure 2D). In response to Na_2_CO_3_, expression peaked at 8 h, fluctuated, and again peaked at 48 h (Figure 2E). Under low-temperature stress, expression was highest at 12 h, gradually declined, and peaked again at 48 h (Figure 2F). In roots, *ZmMYBR24* expression peaked at 4 h post-NaCl treatment, declined, and reached a secondary peak at 12 h (Figure 2G). Under Na_2_CO_3_ stress, expression peaked at 4 h, declined, and began to rise again after 24 h (Figure 2H). Under low-temperature conditions, expression peaked at 4 h (2.12-fold increase), fluctuated, and reached another peak at 48 h (Figure 2I).

**Figure 3:**
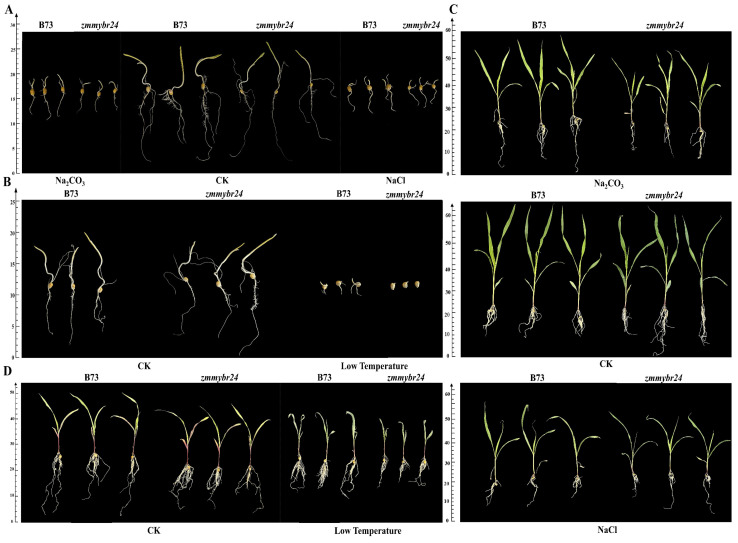
Functional characterization of *ZmMYBR24* under various stress conditions. (**A**) Salinity stress tolerance between wild-type B73 and mutant *zmmybr24* at the germination stage. (**B**) Low-temperature stress tolerance of wild-type B73 and mutant *zmmybr24* at the germination stage. (**C**) Salinity tolerance in seedling wild type B73 and mutant *zmmybr24*. (**D**) Low-temperature tolerance of wild-type B73 and mutant *zmmybr24* at the seedling stage.

To assess stress tolerance, root and shoot growth parameters were compared between wild-type B73 and *zmmybr24* mutant plants under salt, alkali, and low-temperature stress during the germination stage. Root and shoot growth were significantly more inhibited in the mutant line than in B73 under both salt and alkali stress (Figure 3A). The relative germination percentage, germination index, and shoot length were all significantly or highly significantly reduced in the mutant (Table 1). Under low-temperature conditions, the mutant line also exhibited substantially more inhibition of root and shoot growth than B73 (Figure 3B), with significant reductions in shoot length, root length, and root surface area (Table 1).

Similarly, at the seedling stage, *zmmybr24* mutants displayed significantly reduced root and shoot growth under salt and alkali stress compared to wild-type B73 (Figure 3C). The relative seedling fresh weight, root dry weight, and root volume were significantly or highly significantly lower in the mutant (Table 2). Under low-temperature stress, the mutant line again exhibited more severe inhibition of seedling and root growth (Figure 3D), with marked reductions in root length and overall seedling length (Table 2).

**Figure 4:**
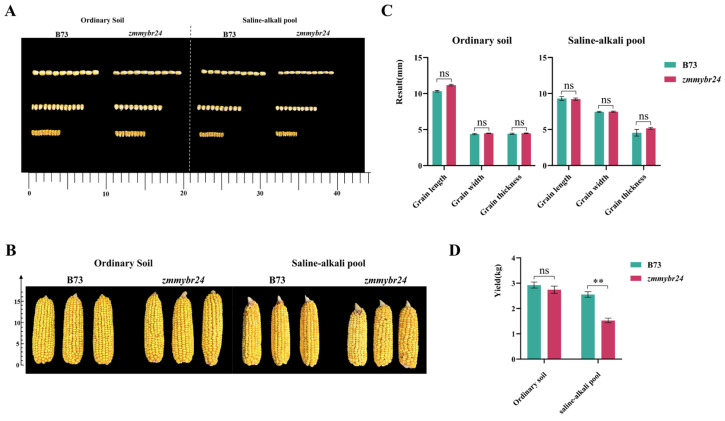
Kernel, ear traits, and yield-related parameters of wild-type B73 and mutant line *zmmybr24* under normal and saline–alkali conditions. (**A**) Seeds of the wild type B73 and the mutant line *zmmybr24*. (**B**) Ears of the wild type B73 and the mutant line *zmmybr24*. (**C**) The differences in kernel length, width, and thickness between wild-type B73 and the mutant line *zmmybr24* grown in ordinary soil versus saline-alkali pool. (**D**) The differences in plot yield between wild-type B73 and the mutant line *zmmybr24* grown in ordinary soil versus saline-alkali pool. Data are presented as the mean ± SD (*n* = 3 independent biological replicates). Statistical significance (*p* < 0.05) was determined by one-way ANOVA with multiple comparisons. ns indicates no significant difference, ** indicate significant differences at *p* < 0.01 levels.

The mutant line *zmmybr24* and the wild-type inbred line B73 were cultivated under both standard field conditions and in a saline–alkali pool to evaluate their performance under stress. Under saline–alkali stress, *zmmybr24* exhibited markedly reduced growth compared to the wild-type B73 (Figure 4A,B). However, when grown under normal field conditions, no significant differences in agronomic or yield-related traits were observed between the two lines (Table 3). Similarly, in the saline–alkali pool, agronomic traits such as grain length, grain width, and grain thickness did not differ significantly between *zmmybr24* and B73 (Figure 4A,C). Despite this, several yield-related traits showed highly significant differences, most notably in plot yield, where *zmmybr24* displayed a substantial reduction (Figure 4D; Table 3).

The resistance functional characterization of the mutant line *zmmybr24* demonstrated that the *ZmMYBR24* gene functions as a positive regulatory transcription factor, conferring resistance to multiple abiotic stresses.

### 2.3. Transcriptome Analysis and Expression Profiling of Key Pathway Genes

**Figure 5:**
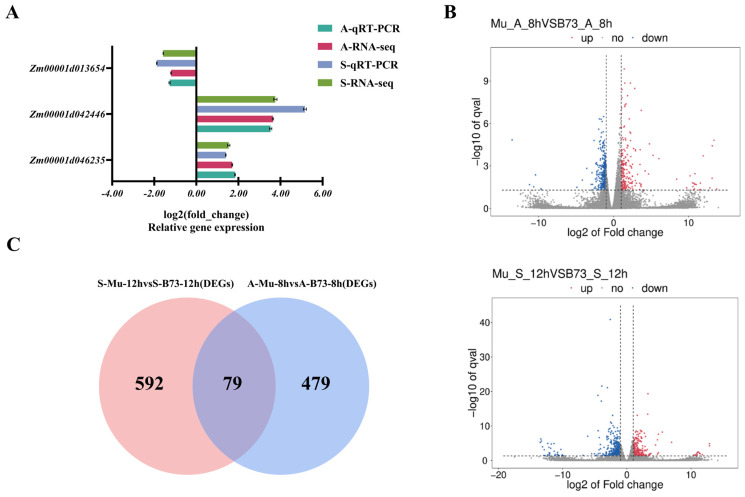
Transcriptome analysis of *zmmybr24* and wild-type B73. (**A**) Validation of RNA-seq results via qRT-PCR. (**B**) Volcano plot showing DEGs between comparison groups. (**C**) Venn diagram of DEGs under salt and alkali stress.

To further explore the molecular mechanisms underlying the stress responses of *ZmMYBR24*, transcriptome sequencing was performed using both the *zmmybr24* mutant and the wild-type B73. Expression patterns of three randomly selected genes were validated via qRT-PCR, and the results were consistent with RNA-seq data, confirming the reliability of the transcriptome analysis (Figure 5A). A comparative analysis revealed that the number of significantly downregulated differentially expressed genes (DEGs) was greater than the number of upregulated DEGs (Figure 5B). Under NaCl and Na_2_CO_3_ stress, 671 and 558 DEGs were identified, respectively (Figure S3). Notably, 79 DEGs were found to be shared across both stress conditions, suggesting common regulatory pathways in response to salt and alkali stress (Figure 5C).

**Figure 6:**
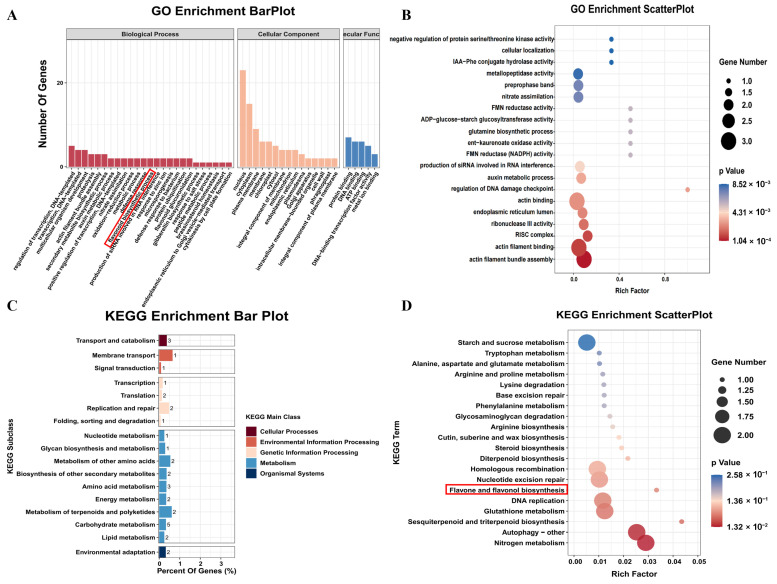
GO and KEGG enrichment analysis of DEGs. (**A**) GO enrichment histogram under NaCl and Na_2_CO_3_ stress. (**B**) GO enrichment scatter plot. (**C**) KEGG pathway histogram. (**D**) KEGG enrichment scatter plot under salt and alkali stress.

GO enrichment was conducted on three distinct DEG sets (*p* < 0.05). Among the 671 DEGs analyzed, the results showed enrichment in 93 biological process terms, 12 cellular component terms, and 42 molecular function terms. For the set of 558 DEGs, 93 biological processes, 29 cellular components, and 63 molecular functions were significantly enriched. In the analysis of the 79 DEGs, enrichment was detected in 108 biological processes, 46 cellular components, and 88 molecular functions. The results were primarily enriched in the flavonoid biosynthetic process (GO:0009813), cellular components such as the nucleus (GO:0005634), and molecular functions including protein binding (GO:0005515) (Figure 6A). At a significance threshold of *p* < 0.05, a total of 38 biological processes, seven cellular components, and 33 molecular functions were significantly enriched. Notable enrichments included nitrate assimilation (GO:0042128), the endoplasmic reticulum lumen (GO:0005788), and phosphatase activity (GO:0004721) (Figure 6B). KEGG pathway enrichment analysis of 671 DEGs (*p* < 0.05) revealed eight significantly enriched pathways, while seven enriched pathways were identified among the 558 DEGs under alkaline stress. Of the 79 DEGs common to both conditions, 36 KEGG pathways were enriched, with plant hormone signal transduction (k04075) being especially prominent (Figure 6C). Furthermore, under the same significance threshold, 2 KEGG pathways were significantly enriched, mainly related to nitrogen metabolism (k00910) and autophagy (k04136) (Figure 6D).

**Figure 7:**
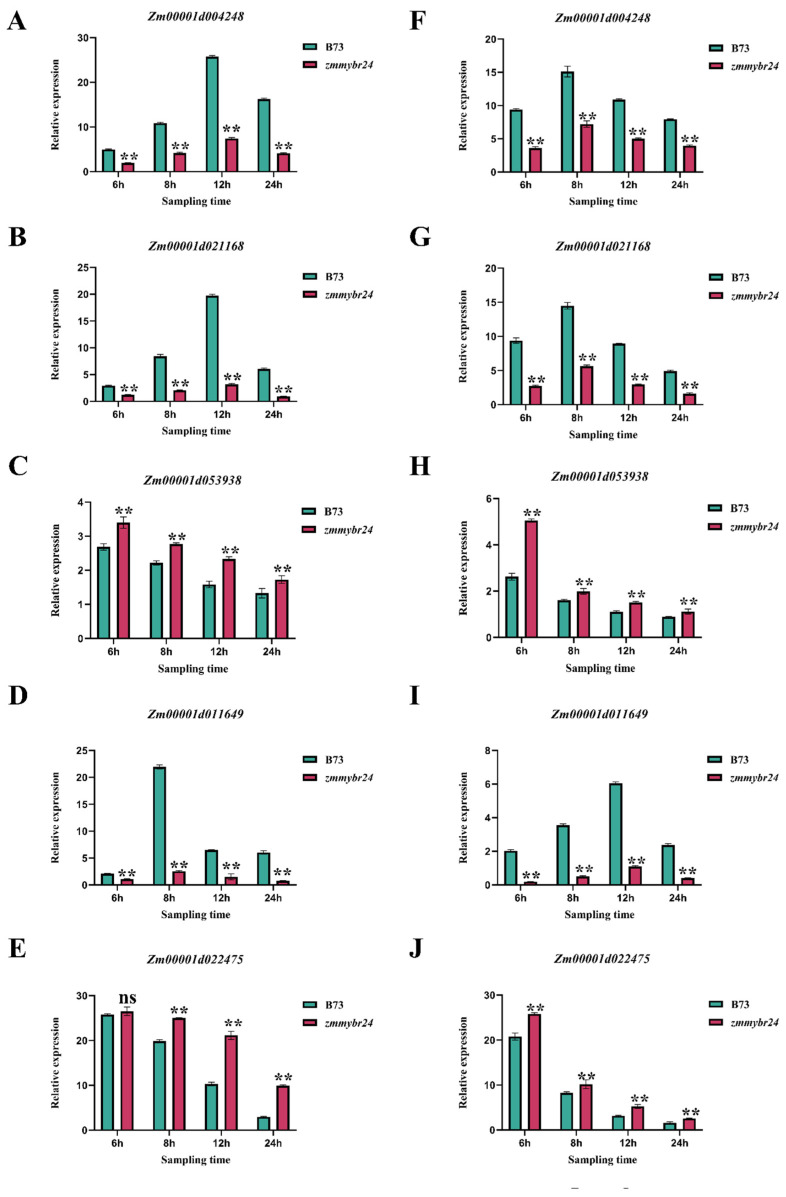
Expression profiling of key pathway genes. (**A**) Relative expression of *Zm00001d004248* gene under NaCl stress. (**B**) Relative expression of *Zm00001d021168* gene under NaCl stress. (**C**) Relative expression of *Zm00001d053938* gene under NaCl stress. (**D**) Relative expression of *Zm00001d011649* gene under NaCl stress. (**E**) Relative expression of *Zm00001d022475* gene under NaCl stress. (**F**) Relative expression of *Zm00001d004248* gene under Na_2_CO_3_ stress. (**G**) Relative expression of *Zm00001d021168* gene under Na_2_CO_3_ stress. (**H**) Relative expression of *Zm00001d053938* gene under Na_2_CO_3_ stress. (**I**) Relative expression of *Zm00001d011649* gene under Na_2_CO_3_ stress. (**J**) Relative expression of *Zm00001d022475* gene under Na_2_CO_3_ stress. Data are presented as the mean ± SD (*n* = 3 independent biological replicates). Statistical significance (*p* < 0.05) was determined by one-way ANOVA with multiple comparisons. ns indicates no significant difference, ** indicate significant differences at *p* < 0.01 levels.

The GO and KEGG analyses collectively indicated that DEGs were predominantly enriched in pathways associated with flavonoid biosynthesis. Preliminary qRT-PCR validation demonstrated that the DEGs in the flavonoid biosynthesis pathway function cooperatively with *ZmMYBR24*. To validate the involvement of these pathways, five flavonoid biosynthesis-related DEGs were selected for qRT-PCR analysis. Under NaCl stress, the expression levels of *Zm00001d004248*, *Zm00001d021168* and *Zm00001d011649* in the mutant *zmmybr24* line were significantly lower than those in the wild-type B73. Both genes showed an early increase in expression, followed by a decline (Figure 7A–C). In contrast, *Zm00001d053938* and *Zm00001d022475* were significantly upregulated in *zmmybr24* compared to B73 (Figure 7D,E). A similar trend was observed under Na_2_CO_3_ stress: *Zm00001d004248*, *Zm00001d021168* and *Zm00001d011649* again exhibited reduced expression in *zmmybr24*, with a transient induction followed by repression (Figure 7F–H), while *Zm00001d053938* and *Zm00001d022475* remained significantly upregulated in the mutant line (Figure 7I,J).

### 2.4. Sequence Variation Analysis of the ZmMYBR24 Gene

**Figure 8:**
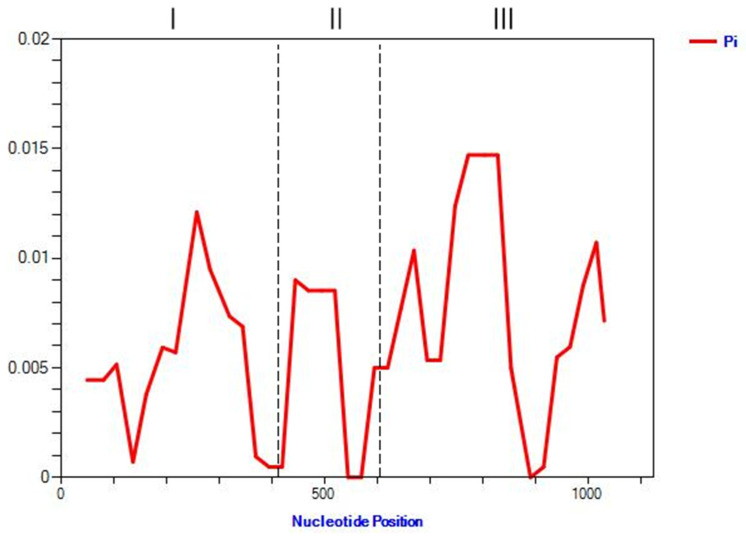
Nucleotide diversity across the coding region of *ZmMYBR24*. Roman numerals indicate exon regions: I (exon 1), II (exon 2), III (exon 3).

To investigate sequence-level diversity, the coding region of *ZmMYBR24* was analyzed in 80 maize inbred lines. Nucleotide diversity (π) peaked at 0.01210 in the first exon and 0.00852 in the second exon, indicating relatively conserved regions. However, a high degree of variation was observed between 724 and 891 bp in the third exon, and that polymorphism is significantly higher than in the first and second exon regions (Figure 8).

A total of 25 SNPs and 17 InDels were identified within the *ZmMYBR24* coding sequence across the 80 lines (Tables S4 and S5), resulting in 16 haplotypes (Table S6) with a polymorphism information content (PIC) of 0.858. Six major haplotypes—HAP1, HAP3, HAP6, HAP9, HAP10, and HAP12—accounted for 80.0% of the total accessions. The superior haplotype HAP12, which included inbred lines such as C649, Dan360, and Dan340, was identified in 8 of the 80 materials (Table 4). Among the SNPs, 12 were synonymous and 13 were nonsynonymous. The nonsynonymous mutations may alter protein structure and function, potentially contributing to phenotypic variation in stress resistance (Table S7).

The nucleotide diversity analysis conducted using DNAsp v6.0 revealed a Tajima’s D value of −1.33391, with haplotype diversity and nucleotide diversity calculated at 0.891 and 0.07312, respectively. None of these parameters reached statistical significance, suggesting that the *ZmMYBR24* gene has undergone neutral evolution within the tested maize population.

## 3. Discussion

### 3.1. ZmMYBR24 Is a Genetic Manipulation Target for Improving Salt, Alkali, and Low-Temperature Tolerance

The *MYB* gene family comprises a group of evolutionarily conserved transcription factors that serve as essential regulators of plant growth, development, and adaptation to environmental stresses. Among major abiotic stresses, salinity, alkalinity, and low temperature are particularly detrimental to maize yield and often occur simultaneously. In our previous studies, we identified *ZmMYBR24* as a positive regulator of maize tolerance to salt, alkali, and cold stress [28,29]. Here, we validated the gene’s function under three stress conditions—160 mmol/L NaCl, 25 mmol/L Na_2_CO_3_, and 10 °C—by assessing phenotypic responses. Distinct phenotypic differences were observed under all three treatments, confirming the gene’s involvement in stress responses.

Temporal expression analysis revealed that, during germination, *ZmMYBR24* expression peaked at 12 h, 8 h, and 4 h following NaCl, Na_2_CO_3_, and low-temperature treatments, respectively. At the seedling stage, the peak expression in leaves occurred at 48 h (NaCl), 8 h (Na_2_CO_3_), and 12 h (low temperature), while, in roots, maximal expression was observed at 12 h, 4 h, and 4 h, respectively, for the same stress treatments. These patterns suggest that *ZmMYBR24* is actively involved in coordinating early and tissue-specific responses to these abiotic stresses. Notably, *ZmMYBR24* expression was significantly higher in the wild-type inbred line B73 than in the *zmmybr24* mutant, indicating that the mutant is more sensitive to stress, whereas B73 exhibits enhanced tolerance. These results demonstrate that *ZmMYBR24* is a positive regulatory transcription factor that enhances tolerance to multiple abiotic stresses by modulating the flavonoid biosynthesis pathway, making it an ideal genetic target for improving maize resistance to saline–alkali and low-temperature stresses. In subsequent studies, the molecular marker-assisted selection (MAS) of SNP loci tightly linked to superior haplotypes will be implemented in combination with relevant phenotypic data to accelerate breeding progress. This gene demonstrates significant potential for breeding stress-resilient maize varieties.

### 3.2. Phenotypic Characterization of Maize Under Salt, Alkali, and Low-Temperature Stress

Previous studies have identified various genes associated with maize salt tolerance. For example, the *ZmSTG1* gene, derived from inbred line 082, showed breeding potential based on enhanced shoot length, shoot fresh weight, and plot yield [30]. Similarly, *ZmNHX5* and *ZmKEA2* were shown to influence salt tolerance through phenotypic traits such as shoot length and dry weight [31]. *ZmSRG7* overexpression significantly improved the germination rate and root length under salt stress, supporting its role in enhancing tolerance [32]. In another study, *ZmbHLH32*-overexpressing lines exhibited alleviated root growth inhibition under NaCl stress, while *zmbhlh32*-CRISPR mutants showed shorter roots compared to the wild-type specimens, confirming *ZmIAA9*’s role in salt tolerance [33]. Moreover, under mild saline–alkali soil conditions, the complementary line expressing *ZmL75* exhibited greater plant height than the control line, establishing *ZmL75* as the causal gene for the salt–alkali sensitivity phenotype *s75* [34].

In the current study, we validated the positive regulatory role of *ZmMYBR24* in maize salt and alkali tolerance in relation to diverse phenotypic metrics, including the germination rate, bud and root length, root dry weight, plant height, and plot yield. Additional indices such as aboveground and root fresh weight, ear height, kernel width, kernel length, and kernel thickness further confirmed *ZmMYBR24*’s contribution to stress tolerance in maize. These results support its utility in maize breeding programs focused on improving resilience to saline, alkaline, and low-temperature environments. In this study, the *zmmybr24* mutant lines showed suppressed phenotypes under multiple abiotic stresses. We hypothesize that *ZmMYBR24* may enhance saline–alkali tolerance by regulating the expression of flavonoid biosynthesis genes to improve ROS scavenging capacity, thereby alleviating oxidative damage.

### 3.3. Transcriptomic Network Construction and Analysis in Maize Under Combined Abiotic Stresses

Plant adaptation to abiotic stress involves a highly complex regulatory network that orchestrates the activity of numerous genes and signaling cascades. Advancements in transcriptome sequencing have significantly enhanced our ability to dissect the molecular basis of these stress responses [35,36]. Under salt and alkali stress, large numbers of DEGs are activated, encompassing a wide array of biological processes. Key regulatory genes—especially transcription factors such as MYB and WRKY—have been identified through co-expression network analysis [37,38]. Among plant transcription factor families, MYBs are notably abundant and functionally diverse, playing critical roles in orchestrating defense mechanisms against abiotic challenges. These factors are frequently situated at the top of regulatory hierarchies and mediate stress responses by controlling the expression of downstream targets [39,40,41].

In this study, transcriptome analysis of the *ZmMYBR24* gene under NaCl stress revealed enrichment in biological processes such as protein phosphorylation, salt stress responses, abscisic acid signaling, and flavonoid biosynthesis. The cellular components that were predominantly enriched included the plasma membrane, cytoplasm, cell wall, and chloroplast. Molecular functions enriched under NaCl stress involved calmodulin binding, nitrate: proton symporter activity, and phosphatase activity. Under Na_2_CO_3_ stress, enriched biological processes included the abscisic acid response, protein phosphorylation, defense response, and salt stress response. The enriched cellular components were similar, encompassing the plasma membrane, cell wall, plasmodesmata, and chloroplasts, while molecular functions involved calcium ion binding, kinase activity, metal ion binding, and transport functions [28,29].

Genes commonly differentially expressed under both stress treatments were significantly enriched in biological processes such as the abscisic acid response, nitrate assimilation, multicellular development, flavonoid biosynthesis, and auxin metabolism. These genes were primarily localized to the nucleus, plasma membrane, cytoplasm, and plasmodesmata, and were associated with molecular functions including protein binding, phosphatase activity, and oxidoreductase activity. These findings highlight the multifaceted role of *ZmMYBR24* in orchestrating maize responses to multiple abiotic stresses and suggest its central position in a broader regulatory network.

We propose that, in the *zmmybr24* mutant under saline–alkali stress, the impaired flavonoid biosynthesis pathway reduces ROS scavenging capacity, leading to increased MDA accumulation, decreased antioxidant enzyme activities, and exacerbated oxidative damage. Conversely, wild-type B73 maintains ROS homeostasis through this pathway, mitigating membrane system injury.

### 3.4. Potential Regulatory Mechanisms of ZmMYBR24 Under Salt and Alkali Stress

To elucidate the functional role of *ZmMYBR24* in maize tolerance to salt and alkali stress, transcriptome (RNA-seq) analysis was conducted to compare gene expression profiles between the wild-type inbred line B73 and the *zmmybr24* mutant under stress conditions. GO enrichment analysis of DEGs revealed significant enrichment in pathways associated with protein serine/threonine kinase activity, protein phosphorylation, responses to abscisic acid, flavonoid biosynthesis, salt stress response, and calcium ion binding—processes integral to the plant’s adaptation to abiotic stress.

These findings suggest that *ZmMYBR24* may regulate the expression of genes involved in flavonoid biosynthesis, enhancing the plant’s antioxidant defense and improving stress resilience. Numerous studies have shown that excessive reactive oxygen species (ROS) accumulation under salt and alkali stress leads to oxidative damage, especially to cell membranes, which is a major constraint on crop growth [42,43,44]. Flavonols are known to enhance the antioxidant capacity of plants, with superoxide dismutase (SOD) catalyzing the conversion of excess ROS to hydrogen peroxide (H_2_O_2_). Flavonoid compounds further mitigate ROS accumulation, thereby improving the plant’s ability to tolerate salt and alkali stress [45,46].

Previous research demonstrated that, under salt and alkali stress, the flavonol synthase gene *MsFLS13* promotes flavonol accumulation in *Medicago sativa* L., reducing ROS content and improving photosynthetic efficiency. This stabilizes the antioxidant system and enhances stress tolerance [47]. Similarly, Feng et al. reported that flavonoids play a key role in salt stress mitigation by scavenging ROS, with the *AcCHI* gene—a critical enzyme in the flavonoid biosynthetic pathway—being implicated in enhanced stress tolerance in forage grasses and crops [48].

In this study, transcriptomic analysis confirmed significant enrichment of the flavonoid biosynthesis pathway under both salt and alkali stress conditions. These findings support the hypothesis that *ZmMYBR24* contributes to stress resistance by modulating the expression of flavonoid biosynthesis-related genes, thereby boosting the plant’s capacity to cope with adverse environmental conditions. The transcriptome analysis results of this study showed significant co-enrichment in the flavonoid biosynthesis pathway under saline–alkali stress. Notably, all genes in this pathway exhibited significant changes in the mutant line. qRT-PCR validation of other flavonoid synthesis genes further suggested potential cooperative regulatory relationships.

## 4. Materials and Methods

### 4.1. Plant Materials

The *zmmybr24* mutant line and the wild-type inbred line B73 were sourced from the Maize EMS-Induced Mutant Library (http://maizeems.qlnu.edu.cn/ (accessed on 20 April 2023)) and used for gene expression analysis, functional validation, and transcriptome sequencing. Additionally, 80 genetically diverse inbred maize lines (Table S8), previously characterized for their tolerance to salt, alkali, and low-temperature stress, were employed for the genetic variation analysis [49,50,51].

### 4.2. Characterization of ZmMYBR24

The fundamental properties of *ZmMYBR24* were analyzed using a suite of bioinformatics tools. Chromosomal location was identified through MaizeGDB (http://www.maizegdb.org/ (accessed on 6 June 2024)), and gene structure was predicted using FGENESH (https://www.softberry.com/berry.phtml (accessed on 6 June 2024)). Cis-regulatory elements in promoter regions were analyzed via PLANTCARE (http://bioinformatics.psb.ugent.be/webtools/plantcare/html/ (accessed on 6 June 2024)). The physicochemical properties of the encoded protein were evaluated using ProtParam (http://web.expasy.org/protparam/ (accessed on 6 June 2024)). Conserved domain analysis was performed with SMART (http://smart.embl.de/ (accessed on 6 June 2024)), while subcellular localization was predicted using PSORT (https://www.genscript.com/psort.html (accessed on 6 June 2024)). Transmembrane structures and signal peptides were predicted using TMHMM v.2.0 (http://www.cbs.dtu.dk/services/TMHMM/ (accessed on 6 June 2024)) and SignalP 4.1 (http://www.cbs.dtu.dk/services/SignalP/ (accessed on 6 June 2024)), respectively. Functional sites were annotated using PROSITE (http://prosite.expasy.org/ (accessed on 6 June 2024)), and phylogenetic analysis was conducted via ClustalW (http://www.ebi.ac.uk/clustalw/ (accessed on 6 June 2024)).

For the yeast two-hybrid (Y2H) assay, the coding sequence of *ZmMYBR24* was cloned into the pGBKT7 vector and co-transformed with pGADT7 into yeast receptor cells. Positive (pGBKT7-53 + pGADT7-T) and negative (pGBKT7-Lam + pGADT7-T) controls were included. Yeast cells were grown on an SD/-Leu/-Trp medium and subsequently transferred to SD/-Leu/-Trp/-His/-Ade/X-α-Gal/AbA (0, 50, 100, 150, 200 ng/mL)/3AT (0, 30, 50, 80 mM) plates for autoactivation testing. Primer sequences are listed in Table S9.

For the low-temperature assays, embryos were collected from two inbred maize lines: the highly tolerant Zao8-3 and the sensitive Ji853. Samples were collected at 0 h, 2 h, and 4 h post-treatment during germination for the qRT-PCR analysis. Similarly, for salt and alkali tolerance tests, embryonic tissues from the stress-tolerant K10 and the sensitive Mo17 lines were harvested at 2, 3, and 4 days after treatment for qRT-PCR.

### 4.3. Characterization of Functional Mutant Lines

The EMS-induced mutant line *zmmybr24* was used as the experimental material, with primers listed in Table S9. After sequence confirmation, homozygous mutant lines were generated through successive backcrossing and self-pollination and subsequently employed in downstream analyses.

Germination stage: seeds were surface-sterilized in 10 g/L sodium hypochlorite for 15 min and rinsed three times with sterile water. They were then soaked for 6 h at room temperature in sterile water, 160 mmol/L NaCl, or 25 mmol/L Na_2_CO_3_. A standard germination test was performed by placing 50 seeds between two layers of moistened filter paper [52], followed by incubation in darkness at 25 °C with 65% ± 5% relative humidity (RH) for 7 days to determine the germination percentage (GP). Root length (RL) was quantified using an Epson scanner and Regent WinRHIZO (Pro 5.0) Canada software. Root fresh weight (RFW) and shoot fresh weight (SFW) were recorded after gently blotting off surface moisture. For the dry weight measurements, seedlings were initially dried at 105 °C for 30 min and then at 80 °C until reaching a constant weight to obtain the root dry weight (RDW) and shoot dry weight (SDW). Shoot length (SL) was measured using a ruler.

For the low-temperature stress treatment, seeds were incubated at 10 °C for 31 days, followed by recovery at 15 °C for 7 days. The control group was maintained at 25 °C for 6 days. To ensure consistent RH (65% ± 5%) under 0 lux dark conditions, distilled water was added daily. GP was assessed on day 31 of treatment, while SL was measured following the recovery phase. RFW, SFW, RDW, SDW, and RL were evaluated for both the treated and control groups, following the criteria outlined in Table S10.

Seedling stage: Seeds were surface-sterilized and soaked as described above and then germinated in a growth chamber at 25 °C (65% ± 5% RH). Uniform seedlings were transplanted into sand-filled pots (five seedlings per pot). Three treatments were applied: control (½-strength Hoagland solution), salt stress (160 mmol/L NaCl + ½ Hoagland), and alkali stress (25 mmol/L Na_2_CO_3_ + ^1^/_2_ Hoagland), with 30 seedlings per treatment. Treatments were administered every two days until leachate appeared, with pre-irrigation performed to prevent salt accumulation. Plants were grown under a 16 h of light/8 h of dark photoperiod (3000 lux, 65% ± 5% RH). After 7 days of treatment, GP, RL, RFW, SFW, RDW, SDW, and SL were assessed using the same methods as in the germination stage.

For low-temperature stress, seedlings at the two-leaf, one-heart stage were subjected to either 25 °C (control) or 4 °C (treatment) for 7 days, with 30 seedlings per condition. Parameters were measured as described above and evaluated based on the standards shown in Table S10.

All phenotypic traits are expressed as relative values: relative germination percentage (RGP), relative root length (RRL), relative root fresh weight (RRFW), relative shoot fresh weight (RSFW), relative root dry weight (RRDW), relative shoot dry weight (RSDW), relative shoot length (RSL), and relative root volume (RRV). These were calculated as the ratio of each trait under stress (salt, alkali, or low temperature) to its corresponding value in the control group. Trait means, standard deviations, and analysis of variance (ANOVA) were computed using IBM SPSS Statistics v26.0.

### 4.4. Expression Pattern Analysis of ZmMYBR24

To analyze the expression dynamics of *ZmMYBR24*, both the mutant line *zmmybr24* and the wild-type inbred line B73 were used at the germination and seedling stages under stress conditions. During germination, embryo samples were collected at 0, 6, 8, 12, 24, 30, and 36 h post-saline or alkali stress treatment, and at 0, 2, 4, 6, 8, and 12 h following low-temperature stress.

During the seedling stage, samples were collected at 0, 4, 8, 12, 24, and 48 h after saline and alkali stress exposure. In addition, root and leaf tissues were harvested at the same time points following both saline and low-temperature treatments. All samples were immediately frozen in liquid nitrogen.

Total RNA was extracted using the AllStyle Gold TransZol Up Plus RNA Kit, (TransGen Biotech Co., LTD, Beijing, China) and the concentration and quality of the extracted RNA were measured using UV spectrophotometry. RNA samples with OD260/280 ratios between 1.8 and 2.0, indicating high quality, were used for subsequent experiments. First-strand cDNA synthesis was performed with an AllStyle Gold TransScript^®^ II Kit, (TransGen Biotech Co., LTD, Beijing, China). qPCR was conducted using a two-step protocol with the primers listed in Table S9. Fluorescent qPCR was carried out using a Jena AG (Jena, Germany) instrument, and relative expression levels were calculated using the 2^−ΔΔCT^ method.

### 4.5. Field Characterization of Salt and Alkali Stress Phenotypes

To assess stress tolerance in field conditions, both *zmmybr24* and the wild-type B73 were grown using a comparative design. Plants were cultivated in both standard soil and saline–alkaline plots using a randomized block design at the transgenic test site of Northeast Agricultural University (126.7° E, 45.7° N; mid-temperate zone). Field management followed standard agronomic practices. Each treatment was replicated three times, with three rows per replication. Rows were spaced 65 cm apart, each 3 m long, with a 25 cm plant interval, maintaining a density of 13 seedlings per row.

The physicochemical properties of the saline–alkaline soil were as follows: soda saline–alkali soil including 108.30 mg/kg available nitrogen, 83.94 mg/kg available phosphorus, 204.6 mg/kg available potassium, and a pH of 8.94. No abnormal climatic variations were detected during the study period. Agronomic traits were evaluated as follows: plant height was measured from ground level to the tip of the tassel; spike height from ground level to the uppermost spike node; and spike length from base to tip. The number of grains per row was recorded for the most uniform ear, and the bald tip length refers to the sterile portion at the spike tip. The 100-grain weight was obtained by randomly sampling and weighing 100 grains. Plot yield was calculated by weighing air-dried, threshed grain samples and standardizing to 14% moisture content.

### 4.6. Transcriptome Analysis and Expression Profiling of Critical Pathway Genes

The mutant line *zmmybr24* and the wild-type inbred line B73 were used as experimental materials. Embryonic tissues were sampled during germination at 0 h (unstressed control), 8 h, and 12 h following saline or alkaline stress treatments. Each time point included three biological replicates. Samples were promptly labeled, snap-frozen in liquid nitrogen, and stored for further analysis. Three comparative groups were established between *zmmybr24* and wild-type B73 under distinct conditions: 8 h after alkaline stress (A-Mu-8 h vs. A-B73-8 h), 12 h after salt stress (S-Mu-12 h vs. S-B73-12 h), and an untreated control (Mu-0 h vs. B73-0 h). Differentially expressed genes (DEGs) were identified in each group based on the criteria |log_2_FC| ≥ 1 and *p* < 0.05.

RNA sequencing was conducted by LC-Bio Technologies Co., Ltd. (Hangzhou, China). The sequencing depth was 6G raw data per sample, generated on the Illumina platform, and the RNA-seq libraries were constructed as strand-specific libraries. Sequencing reads were aligned and assembled using TopHat and Cufflinks. DEGs were detected using DESeq, applying thresholds of fold change ≥ 2 (|log_2_FC| ≥ 1) and *q* < 0.05 (where *q* denotes the FDR-adjusted *p*-value). Genes satisfying these criteria were subjected to enrichment analysis and gene set enrichment analysis (GSEA). To validate the RNA-seq results, three DEGs were randomly selected and their expression levels assessed via qRT-PCR. The primer sequences used are listed in Table S9.

GO enrichment analysis was performed to identify all enriched GO terms associated with DEGs in the *ZmMYBR24* gene. The functional categorization of DEGs was based on GO annotations from the V4 public database. Significant GO terms related to *ZmMYBR24* were determined using a hypergeometric test in conjunction with the Phytozome5 database. The functional classification of GO terms was organized into three principal domains: biological processes (BP), cellular components (CC), and molecular functions (MF). To further explore the roles of differentially expressed genes (DEGs), KEGG pathway enrichment analysis was conducted. Gene annotations were cross-referenced using Maize GDB and NCBI databases for comprehensive interpretation.

Three key genes—*Zm00001d004248*, *Zm00001d021168*, *Zm00001d011649*, *Zm00001d022475* and *Zm00001d053938*—were selected from the transcriptome data for the expression analysis. Using *zmmybr24* and B73 as materials, qRT-PCR was performed at 6, 8, 12, and 20 h after saline–alkaline stress during germination. The maize *Actin* gene served as an internal control. Primer details are provided in Table S9.

### 4.7. Sequence Variation Analysis of the ZmMYBR24 Gene

A total of 80 inbred maize lines exhibiting diverse tolerance to salt, alkali, and low temperatures were analyzed. After germination and growth to the three-leaf stage, genomic DNA was extracted using the CTAB method. The *ZmMYBR24* gene, comprising three exons, was amplified using overlapping primers designed with Primer 5.0 (primer sequences in Table S9). High-fidelity Q5 DNA polymerase was used in 50 µL reactions. Five microliters of each product were analyzed via agarose gel electrophoresis; the remaining product was submitted to Beijing Coolaber Technology Co., Ltd. (Beijing, China) for sequencing. Coding sequences (CDS) were extracted using SnapGene 7.2.0 software. Multiple sequence alignment, nucleotide diversity analysis, and haplotype analysis of the *ZmMYBR24* CDS across the 80 lines were performed using DnaSP v6.0. Tajima’s D test was used to assess neutrality.

### 4.8. Statistical Analysis

Data are presented as the mean ± SD (*n* = 3 independent biological replicates). Statistical significance (*p* < 0.05) was determined via one-way ANOVA with multiple comparisons using IBM SPSS Statistics v26.0. ns indicates no significant difference, * and ** indicate significant differences at *p* < 0.05 and *p* < 0.01 levels, respectively.

## 5. Conclusions

In this study, the MYB transcription factor gene *ZmMYBR24* was found to be significantly upregulated under salt, alkali, and low-temperature stress. Functional analysis revealed that the *zmmybr24* mutant exhibited greater inhibition of root and seedling growth compared to the wild-type B73 under these stress conditions. RNA-seq–based GO and KEGG analyses indicated that *ZmMYBR24* may regulate flavonoid biosynthesis genes involved in multi-stress responses, with qRT-PCR confirming differential expression patterns. A sequence analysis of 80 inbred maize lines identified 16 haplotypes, among which HAP12 conferred enhanced resistance to all three stresses. Subsequent studies could utilize marker-assisted selection to introgress the *ZmMYBR24* gene from superior haplotypes into important inbred lines requiring stress tolerance improvement through backcross breeding, thereby accelerating the breeding process and developing new maize varieties. These results provide critical insight into the molecular mechanisms of abiotic stress tolerance in maize and offer a valuable foundation for breeding stress-resilient cultivars.

## Figures and Tables

**Table 1:** Relative values of NaCl, Na_2_CO_3_, and post-low temperature stress trait surveys at the germination stage.

Treatment	NaCl		Na_2_CO_3_		Low Temperature	
Lines	B73	*zmmybr24*	B73	*zmmybr24*	B73	*zmmybr24*
RGP	0.53 ± 0.24	0.21 ± 0.78 **	0.33 ± 0.23	0.08 ± 0.23 *	0.23 ± 0.34	0.13 ± 0.21 *
RSL	0.11 ± 0.88	0.17 ± 0.55 *	0.33 ± 0.05	0.27 ± 0.45 **	0.15 ± 1.01	0.09 ± 0.34 **
RSFW	0.17 ± 0.91	0.12 ± 0.93	0.25 ± 0.08	0.21 ± 0.11	0.07 ± 0.01	0.05 ± 0.34 *
RSDW	0.13 ± 0.26	0.14 ± 0.28	0.32 ± 0.22	0.18 ± 0.12	0.21 ± 0.01	0.14 ± 0.23 *
RRL	0.40 ± 0.44	0.29 ± 0.11 *	0.39 ± 0.27	0.28 ± 0.21 *	0.13 ± 0.24	0.08 ± 0.34 *
RRFW	0.50 ± 0.54	0.34 ± 0.34 *	0.50 ± 0.11	0.35 ± 0.34	0.46 ± 0.33	0.32 ± 0.65 **
RRDW	0.21 ± 0.56	0.20 ± 0.67	0.28 ± 0.01	0.34 ± 0.48	0.21 ± 0.36	0.07 ± 0.34 **

Note: The measured parameters included: relative germination percentage (RGP), relative shoot length (RSL), relative shoot fresh weight (RSFW), relative shoot dry weight (RSDW), relative root length (RRL), relative root fresh weight (RRFW), and relative root dry weight (RRDW). Data are presented as mean ± SD (*n* = 3 independent biological replicates). Statistical significance (*p* < 0.05) was determined by one-way ANOVA with multiple comparisons. ns indicates no significant difference, * and ** indicate significant differences at *p* < 0.05 and *p* < 0.01 levels, respectively.

**Table 2:** Relative values of NaCl, Na_2_CO_3_, and post-low-temperature stress trait surveys at the seedling stage.

Treatment	NaCl		Na_2_CO_3_		Low Temperature	
Lines	B73	*zmmybr24*	B73	*zmmybr24*	B73	*zmmybr24*
RRL	0.92 ± 0.21	0.87 ± 0.53	0.92 ± 1.01	0.81 ± 0.35 **	0.78 ± 0.01	0.66 ± 0.01 *
RRFW	0.79 ± 0.56	0.55 ± 0.23 *	0.77 ± 0.56	0.49 ± 0.56 **	0.78 ± 0.01	0.66 ± 0.01 *
RRDW	0.68 ± 0.02	0.55 ± 0.22 *	0.71 ± 0.89	0.54 ± 0.58 **	0.78 ± 0.01	0.66 ± 0.03 *
RRAD	0.76 ± 0.25	0.67 ± 0.23	0.85 ± 0.07	0.70 ± 0.27 *	0.78 ± 0.02	0.66 ± 0.01 *
RRV	0.75 ± 0.02	0.62 ± 0.35 *	0.77 ± 0.02	0.64 ± 0.57 **	0.78 ± 0.08	0.66 ± 0.03 *
RSL	0.88 ± 0.01	0.86 ± 0.34 *	0.91 ± 0.02	0.87 ± 0.35 **	0.72 ± 0.02	0.64 ± 0.45 **
RSFW	0.66 ± 0.46	0.52 ± 0.56 *	0.70 ± 0.04	0.57 ± 0.71 *	0.76 ± 0.34	0.50 ± 0.65 **
RSDW	0.63 ± 0.34	0.52 ± 0.82	0.63 ± 0.01	0.56 ± 1.01 *	0.91 ± 0.47	0.54 ± 0.26 **

Note: the measured parameters included: relative root length (RRL), relative root fresh weight (RRFW), relative root dry weight (RRDW), relative root average diameter (RRAD), relative root volume (RRV), relative shoot length (RSL), relative shoot fresh weight (RSFW), and relative shoot dry weight (RSDW). Data are presented as the mean ± SD (*n* = 3 independent biological replicates). Statistical significance (*p* < 0.05) was determined by one-way ANOVA with multiple comparisons. ns indicates no significant difference, * and ** indicate significant differences at *p* < 0.05 and *p* < 0.01 levels, respectively.

**Table 3:** Agronomic traits of mutant *zmmybr24* and wild-type B73.

Treatment	Ordinary Soil		Saline-Alkali Pool	
Lines	B73	*zmmybr24*	B73	*zmmybr24*
Plant height (cm)	229.22 ± 0.84	230.89 ± 3.98	218.44 ± 5.97	215.22 ± 4.53
Ear height (cm)	107.78 ± 3.27	105.22 ± 1.35	91.56 ± 184	90.44 ± 0.84
Ear length (cm)	16.82 ± 0.62	16.55 ± 0.37	16.09 ± 0.08	14.11 ± 0.16 **
Rows grains	36.2 ± 0.20	29.53 ± 4.21	24.96 ± 0.71	23.38 ± 0.41 *
Bald tip length(cm)	0.21 ± 0.09	0.25 ± 0.19	1.28 ± 0.02	1.37 ± 0.01 **
100-grain weight (g)	24.96 ± 2.48	24.40 ± 2.64	28.52 ± 0.01	27.02 ± 0.52 **
Yield (kg)	2.79 ± 0.27	2.73 ± 0.08	2.55 ± 0.01	1.54 ± 0.01 **

Note: Data are presented as the mean ± SD (*n* = 3 independent biological replicates). Statistical significance (*p* < 0.05) was determined by one-way ANOVA with multiple comparisons. ns indicates no significant difference, * and ** indicate significant differences at *p* < 0.05 and *p* < 0.01 levels, respectively.

**Table 4:** Inbred lines aligning with superior haplotypes.

Haplotype	Materials
HAP12	C649, 502, LX9801, Liao540, Liao184, Dan360, Dan340, Ji81162

## Data Availability

The transcriptome sequencing data have been deposited in the Sequence Read Archive (SRA) at the National Center for Biotechnology Information (NCBI) under the accession number PRJNA1270777.

## Supplementary Materials

The following supporting information can be downloaded at: https://www.mdpi.com/article/10.3390/plants14132054/s1, Figure S1. Prediction of the functional site of the *ZmMYBR24*. Figure S2. Identification of mutation sites in *ZmMYBR24*. Figure S3. Differentially expressed genes under NaCl stress and Na_2_CO_3_ stress. Figure S4. GO Enrichment Analysis Under Salt and Alkali Stress Conditions. (A) GO enrichment histogram of DEGs under NaCl stress. (B) GO enrichment point diagram of DEGs under NaCl stress. (C) GO enrichment histogram of DEGs under Na_2_CO_3_ stress. (D) GO enrichment point diagram of DEGs under Na_2_CO_3_ stress. Figure S5. KEGG Enrichment Analysis Under Salt and Alkali Stress Conditions. (A) KEGG histogram of DEGs under NaCl stress. (B) KEGG enrichment point diagram of DEGs under NaCl stress. (C) KEGG enrichment point diagram of DEGs under Na_2_CO_3_ stress. (D) Scatter plot of KEGG enrichment under Na_2_CO_3_ stress. Table S1. *Zm00001d008808* transcription factor protein for sequence alignment. Table S2. The results of agronomic traits of mutant zmmybr24 and wild type B73. Table S3. The results of yield traits of mutant *zmmybr24* and wild type B73. Table S4. SNPs and haplotypes of the CDS region of the *ZmMYBR24.* Table S5. InDels and haplotypes of the CDS region of the *ZmMYBR24.* Table S6. Candidate gene *ZmMYBR24* haplotype corresponds to inbred lines. Table S7. changes of amino acids corresponding to SNP of *ZmMYBR24* gene. Table S8. 80 inbred lines in this study. Table S9. The primers used in this study. Table S10. Methods for evaluation of target traits.
