# Supplementary material for: The Transcription Factor ZmMYBR24 Gene Is Involved in a Variety of Abiotic Stresses in Maize (Zea mays L.)
Source: Plants (Basel). 2025 Jul 4;14(13):2054. doi: 10.3390/plants14132054 (PMC12251797; doi:10.3390/plants14132054)
Supplement: Supplementary file 1 [file plants-14-02054-s001.zip › Figure S.pdf]

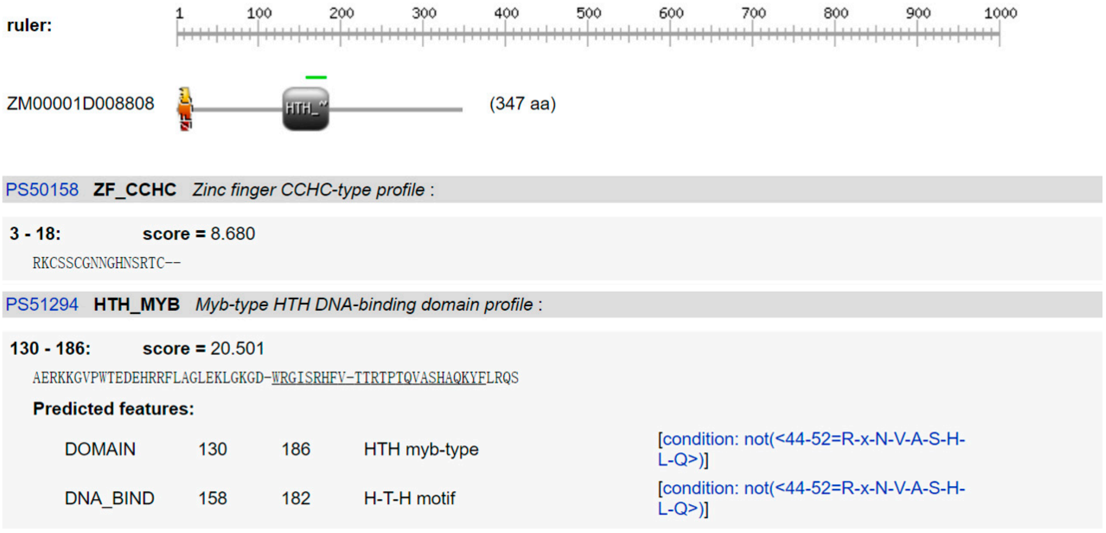

Figure S1 Prediction of the functional site of the *ZmMYBR24*

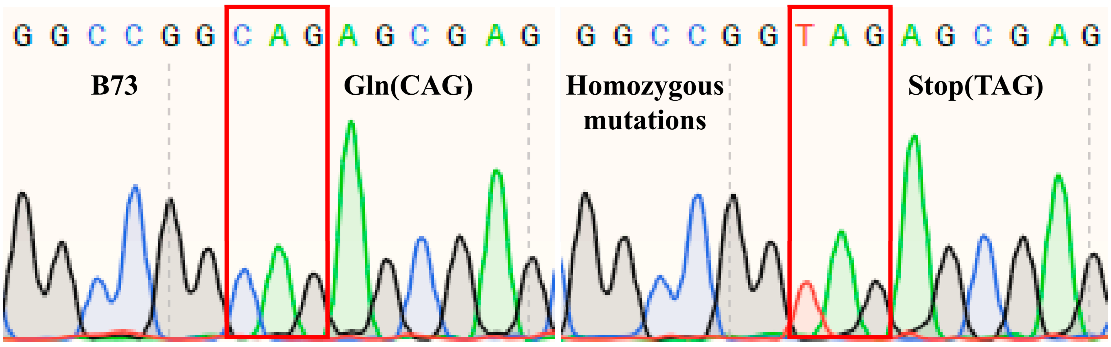

Figure S2 Identification of mutation sites in *ZmMYBR24*

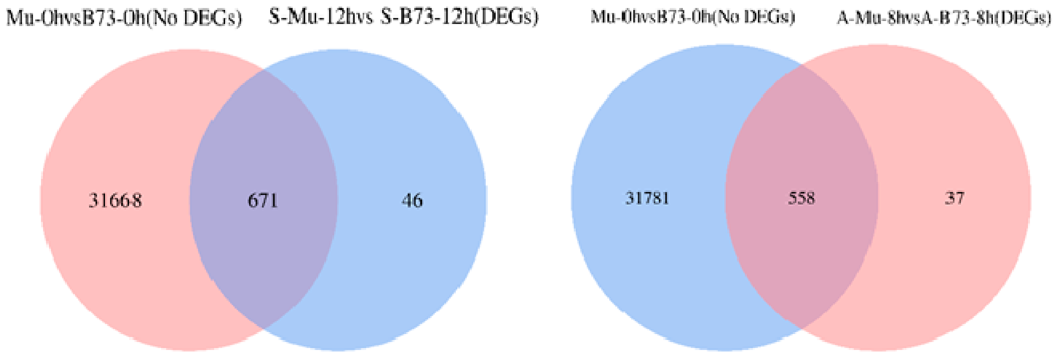

Figure S3 Differentially expressed genes under NaCl stress and Na_2CO_3 stress

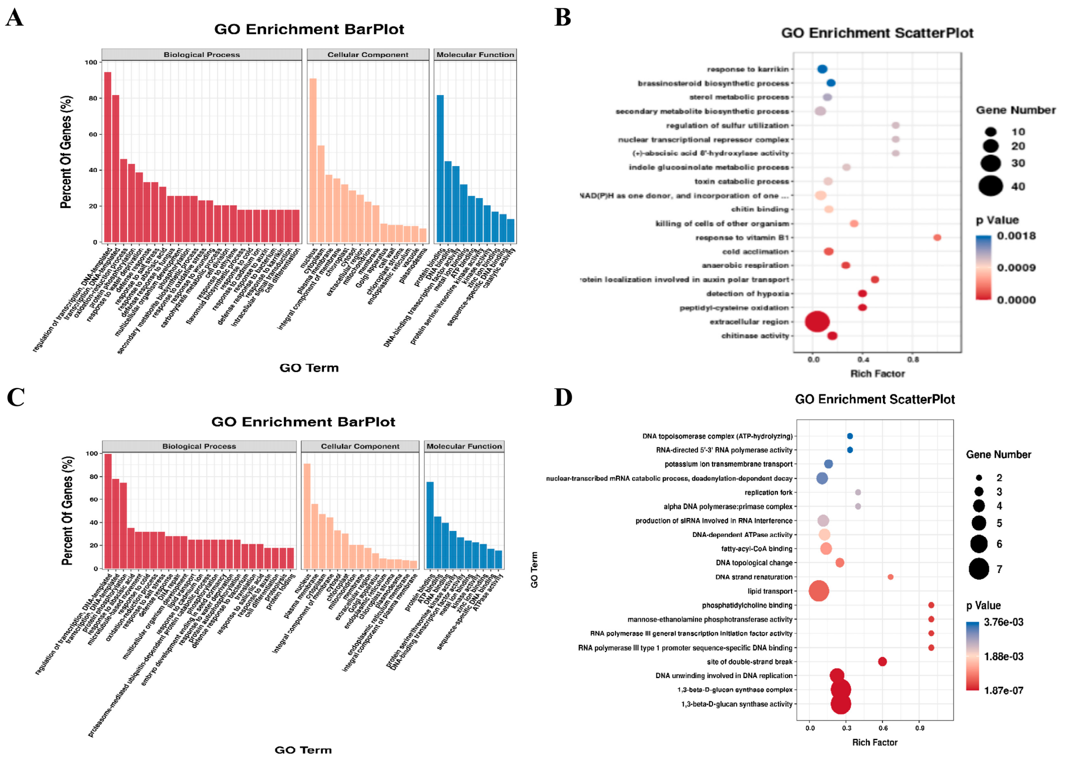

Figure S4 GO Enrichment Analysis Under Salt and Alkali Stress Conditions. (A) GO enrichment histogram of DEGs under NaCl stress. (B) GO enrichment point diagram of DEGs under NaCl stress. (C) GO enrichment histogram of DEGs under Na_2CO_3 stress. (D) GO enrichment point diagram of DEGs under Na_2CO_3 stress.

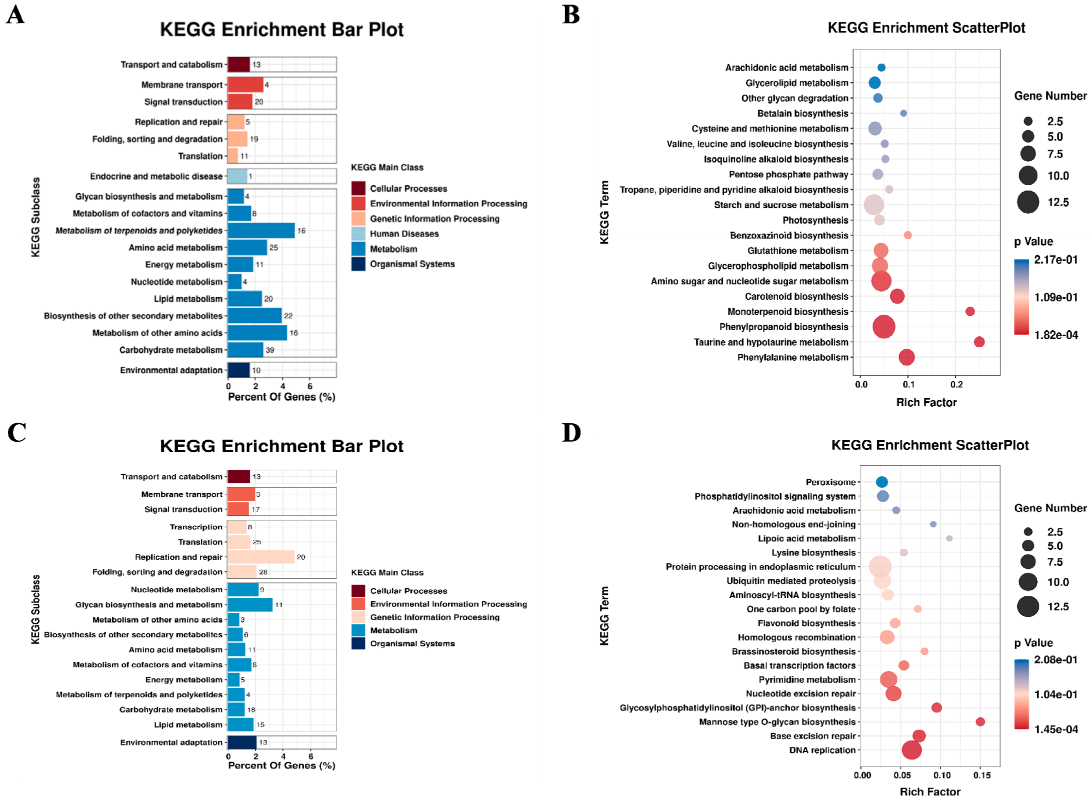

Figure S5 KEGG Enrichment Analysis Under Salt and Alkali Stress Conditions. (A) KEGG histogram of DEGs under NaCl stress. (B) KEGG enrichment point diagram of DEGs under NaCl stress. (C) KEGG enrichment point diagram of DEGs under Na_2CO_3 stress. (D) Scatter plot of KEGG enrichment under Na_2CO_3 stress.
