# Supplementary material for: The Transcription Factor ZmMYBR24 Gene Is Involved in a Variety of Abiotic Stresses in Maize (Zea mays L.)
Source: Plants (Basel). 2025 Jul 4;14(13):2054. doi: 10.3390/plants14132054 (PMC12251797; doi:10.3390/plants14132054)
Supplement: Supplementary file 1 [file plants-14-02054-s001.zip › Table S.pdf]

Table S1 *Zm00001d008808* transcription factor protein for sequence alignment

Code	Gene bank ID	Species	Similarity(%)
Zea maysXP_008655699.1	XP_008655699.1	*Zea mays* L.	100
Zea maysXP_020398412.1	XP_020398412.1	*Zea mays* L.	93.03
Zea maysPWZ09722.1	PWZ09722.1	*Zea mays* L.	85.23
Sorghum bicolorXP_002455021.1	XP_002455021.1	*Sorghum bicolor* L.	63.16
Miscanthus lutarioripariusCAD6233184.1	CAD6233184.1	*Miscanthus lutarioriparius* L. Liu ex Renvoize	71.83
Zea maysNP_001341106.1	NP_001341106.1	*Zea mays* L.	62.53
Saccharum hybrid cultivar R570AGT16142.1	AGT16142.1	*Saccharum hybrid cultivar R570*	68.52
Panicum halliiXP_025818314.1	XP_025818314.1	*Panicum hallii*	56.9
Setaria italicaXP_014660810.1	XP_014660810.1	*Setaria italica*	56
Dichanthelium oligosanthesOEL23995.1	OEL23995.1	*Dichanthelium oligosanthes*	58.24
Digitaria exilisKAF8705725.1	KAF8705725.1	*Digitaria exilis*	58.17
Panicum miliaceumRLN23011.1	RLN23011.1	*Panicum miliaceum*	57.18
Panicum hallii var. halliiPUZ58461.1	PUZ58461.1	*Panicum hallii var. hallii*	56.32
Digitaria exilisCAB3475917.1	CAB3475917.1	*Digitaria exilis*	56.66
Panicum virgatumXP_039846151.1	XP_039846151.1	*Panicum virgatum*	57.02
Eragrostis curvulaTVU21412.1	TVU21412.1	*Eragrostis curvula*	57.67
Digitaria exilisCAB3471894.1	CAB3471894.1	*Digitaria exilis*	57.83
Dimitria exilisKAF8663233.1	KAF8663233.1	*Digitaria exilis*	54.64
Panicum virgatumKAG2586427.1	KAG2586427.1	*Panicum virgatum*	56.4
Oryza brachyanthaXP_006643865.1	XP_006643865.1	*Oryza brachyantha*	51.97
Oryza meyeriana var. granulataKAF0916002.1	KAF0916002.1	*Oryza meyeriana var. granulata*	52.39
Oryza sativa Indica GroupEAY72864.1	EAY72864.1	*Oryza sativa Indica Group*	55.01
Oryza sativaKAB8080324.1	KAB8080324.1	*Oryza sativa*	54.39
Zizania palustrisKAG8045952.1	KAG8045952.1	*Zizania palustris*	48.14

Hordeum vulgare subsp. vulgareBAJ99046.1	BAJ99046.1	*Hordeum vulgare subsp. vulgare*	51.56
Triticum aestivumAIM18728.1	AIM18728.1	*Triticum aestivum*	50.85
Oryza sativa Japonica GroupXP_015625837.1	XP_015625837.1	*Oryza sativa Japonica Group*	54.11
Panicum virgatumKAG2586428.1	KAG2586428.1	*Panicum virgatum*	55.77
Triticum aestivumKAF7028217.1	KAF7028217.1	*Triticum aestivum*	51.44
Triticum dicoccoidesXP_037404461.1	XP_037404461.1	*Triticum dicoccoides*	50.85
Aegilops tauschii subsp. strangulataXP_020153631.2	XP_020153631.2	*Aegilops tauschii subsp. strangulata*	51
Triticum aestivumXP_044355642.1	XP_044355642.1	*Triticum aestivum*	51
Triticum aestivumXP_044347526.1	XP_044347526.1	*Triticum aestivum*	51.44
Triticum dicoccoidesXP_037411743.1	XP_037411743.1	*Triticum dicoccoides*	51.44
Eleusine coracana subsp. coracanaGJN05550.1	GJN05550.1	*Eleusine coracana subsp. coracana*	56.7
Hordeum vulgare subsp. vulgareXP_044975013.1	XP_044975013.1	*Hordeum vulgare subsp. vulgare*	49.01
Setaria viridisXP_034583877.1	XP_034583877.1	*Setaria viridis*	46.54
Setaria italicaXP_004960912.1	XP_004960912.1	*Setaria italica*	47.09
Dichanthelium oligosanthesOEL25569.1	OEL25569.1	*Dichanthelium oligosanthes*	44.32
Lolium rigidumXP_047054758.1	XP_047054758.1	*Lolium rigidum*	47.44
Digitaria exilisCAB3460463.1	CAB3460463.1	*Digitaria exilis*	46.37
Panicum virgatumXP_039836874.1	XP_039836874.1	*Panicum virgatum*	44.32
Brachypodium distachyonXP_003564752.1	XP_003564752.1	*Brachypodium distachyon*	64.59
Panicum virgatumXP_039799445.1	XP_039799445.1	*Panicum virgatum*	44.32
Panicum miliaceumRLN30637.1	RLN30637.1	*Panicum miliaceum*	44.08
Sorghum bicolorKAG0517290.1	KAG0517290.1	*Sorghum bicolor* L.	43.45
Panicum miliaceumRLN00050.1	RLN00050.1	*Panicum miliaceum*	43.49
Oryza brachyanthaXP_006654137.1	XP_006654137.1	*Oryza brachyantha*	52.67
Miscanthus lutarioripariusCAD6272621.1	CAD6272621.1	*Miscanthus lutarioriparius*	44.82
Ananas comosusXP_020087700.1	XP_020087700.1	*Ananas comosus*	55.61

Table S2 The results of agronomic traits of mutant zmmybr24 and wild type B73

Treatment	Ordinary soil		saline pool	
lines	B73	*zmmybr24*	B73	*zmmybr24*
Plant height（cm）	229.22±0.84	230.89±3.98	218.44±5.97	215.22±4.53
Ear height（cm）	107.78±3.27	105.22±1.35	91.56±184	90.44±0.84
Tassel branch number	7.11±0.84	7.11±0.69	5.89±0.51	5.78±0.70

Table S3 The results of yield traits of mutant *zmmybr24* and wild type B73

Treatment	Ordinary soil		saline pool	
lines	B73	*zmmybr24*	B73	*zmmybr24*
Ear length (cm)	16.82±0.62	16.55±0.37	16.09±0.08	14.11±0.16**
Ear diameter (mm)	45.10±1.11	44.16±0.68	43.25±0.01	44.34±1.13
Cob diameter (mm)	23.83±0.96	25.49±1.80	23.99±0.01	25.54±2.07
Grain length(mm)	10.45±1.01	11.11±0.32	9.21±0.01	9.21±0.48
Grain width(mm)	4.41±0.96	4.49±0.12	7.47±0.01	7.47±0.48
Grain thickness(mm)	4.41±0.96	4.49±0.12	4.58±0.01	5.10±0.88
Ear rows	16.53±1.29	15.33±0.46	16.67±0.01	16.00±1.33
Rows grains	36.2±0.20	29.53±4.21	24.96±0.71	23.38±0.41*
Bald tip length(cm)	0.21±0.09	0.25±0.19	1.28±0.02	1.37±0.01**
100-grain weight (g)	24.96±2.48	24.40±2.64	28.52±0.01	27.02±0.52**
Yield (kg)	2.79±0.27	2.73±0.08	2.55±0.01	1.54±0.0**

Note: * and ** indicate significance at $P < 0.05$ and $P < 0.01$, respectively.

Table S4 SNPs and haplotypes of the CDS region of the *ZmMYBR24*

Haplotype	SNP position																							
	0	0	0	0	0	0	0	0	0	0	0	0	0	0	0	0	0	0	0	0	1	1	1	1	1
	0	1	2	2	2	2	2	3	3	4	4	4	6	6	7	7	8	8	9	9	0	0	0	0	0
	7	5	0	1	4	5	9	0	4	1	8	9	2	9	0	8	0	1	5	6	0	1	4	4	5
	9	3	1	1	0	9	0	0	5	7	0	3	1	4	6	8	3	4	4	9	2	9	1	7	1
HAP1	G	G	G	C	C	G	C	G	A	G	C	A	A	G	G	C	A	C	T	G	G	A	G	C	C
HAP2	G	G	G	T	C	G	C	C	A	G	G	C	A	G	G	C	A	C	T	G	G	A	G	C	C
HAP3	A	G	G	T	C	G	C	C	A	G	G	C	A	G	G	C	A	C	T	G	G	A	G	C	C
HAP4	A	A	G	T	C	G	C	G	A	G	C	A	A	G	G	C	A	C	T	G	G	A	G	C	C
HAP5	A	G	G	C	C	A	C	G	T	G	C	A	A	G	G	C	A	C	T	G	G	A	G	C	C
HAP6	A	G	G	T	C	G	C	C	A	G	C	A	A	G	G	C	A	C	T	G	G	A	G	C	C
HAP7	A	G	G	T	C	G	C	C	A	T	G	A	A	G	G	C	A	C	T	G	G	A	G	C	C
HAP8	A	G	C	T	C	G	C	C	A	G	G	C	A	G	G	C	A	C	T	G	G	A	G	C	C
HAP9	G	G	G	T	C	G	C	C	A	G	G	C	T	G	G	T	G	A	T	A	G	A	G	C	C
HAP10	A	G	G	T	C	G	C	C	A	G	G	C	T	G	G	T	G	A	T	A	G	A	G	C	C
HAP11	G	G	G	C	C	G	C	G	A	G	C	A	A	C	C	T	G	C	C	G	G	C	A	A	C
HAP12	A	G	G	T	A	G	T	G	A	G	G	C	A	C	C	T	G	C	T	G	G	C	G	C	A
HAP13	A	G	G	T	A	G	T	G	A	G	G	C	A	C	C	T	G	C	C	G	G	C	A	A	C
HAP14	A	G	G	T	A	G	C	G	A	G	G	C	A	C	C	T	G	C	T	G	A	A	G	C	C
HAP15	A	G	G	T	C	G	C	C	A	G	G	C	A	C	C	T	G	C	T	G	G	C	G	C	A
HAP16	A	G	G	T	C	G	C	C	A	G	G	C	A	C	C	T	G	C	T	G	G	G	C	C	C

Table S5 InDels and haplotypes of the CDS region of the *ZmMYBR24*

Haplotype	InDels position															
	73-78	107-109	113-115	175-180	222-223	224-229	230-232	233-234	235	236	291-296	306-311	721-723	781-786	856-861	871-876	1042-1044
HAP1	/	/	/	/	/	/	/	/	/	/	/	/	/	/	/	/	/
HAP2	/	/	/	/	-GC	-TGCCGC	-CGC	-CG	-C	-C	/	/	/	/	+GGCGTC	/	/
HAP3	/	+CCG	/	/	/	-TGCCGC	/	/	/	/	/	/	/	/	/	/	/
HAP4	/	/	/	/	-CC	-TGCCGC	-CGC	-CG	-C	-C	/	/	/	/	/	/	/
HAP5	+ACGGCC	/	/	/	/	/	/	/	/	/	/	-GGCGTC	/	/	/	/	/
HAP6	/	+CCG	/	/	-GC	-TGCCGC	-CGC	-CG	-C	-C	/	/	/	/	/	/	/
HAP7	/	+CCG	/	/	-GC	-TGCCGC	-CGC	-CG	-C	-C	/	/	/	/	/	/	/
HAP8	/	+CCG	/	/	-GC	-TGCCGC	-CGC	-CG	-C	-C	/	/	/	/	/	/	/
HAP9	/	/	/	/	-GC	-TGCCGC	-CGC	-CG	-C	-C	/	/	-ACG	+CTTGAC	+GGCGTC	/	/
HAP10	/	+CCG	/	/	-GC	-TGCCGC	-CGC	-CG	-C	-C	/	/	-ACG	+CTTGAC	+GGCGTC	/	/
HAP11	/	/	/	/	/	/	/	/	/	/	/	/	/	+CTTGA	+GGCGT	+TCGCC	/

														C	C	G	
HAP12	/	/	+GCG	+CCCATG	/	-TGCCGC	-CGC	/	T	/	-GTCGGC	/	/	+CTTGAC	+GGCGTC	+TCGCCG	/
HAP13	/	/	+GCG	+CCCATG	/	/	-CGC	/	T	/	-GTCGGC	/	/	+CTTGAC	+GGCGTC	+TCGCCG	/
HAP14	/	/	+GCG	+CCCATG	/	-TGCCGC	-CGC	/	T	/	/	-GGCGTC	/	+CTTGAC	+GGCGTC	+TCGCCG	+ACG
HAP15	/	+CCG	/	/	-GC	-TGCCGC	/	-CG	-C	/	/	/	/	+CTTGAC	+GGCGTC	+TCGCCG	/
HAP16	/	+CCG	/	/	-GC	-TGCCGC	/	-CG	-C	/	/	/	/	+CTTGAC	+GGCGTC	+TCGCCG	+ACG

Table S6 Candidate gene *ZmMYBR24* haplotype corresponds to inbred lines

Haplotype	No. of lines	Lines
HAP1	11	Jinhuang55、Ji465、J001、CAL70、Dong46、Chang3、Ji63、Si387、6523、32、Jinhuang63
HAP2	3	Y7、Jan-65、Jinhuang73
HAP3	5	Ji412、K10、Suixi707、B84、Qing795
HAP4	3	77、706Fu、888-9
HAP5	2	Chong72、8129
HAP6	6	Guan17、Ji846、803、Zong31、7884、DH34
HAP7	2	D387、Zhongzong4C1-3-2-2-b-5-2-1-b
HAP8	1	Si-279
HAP9	11	SH15、Chuan273、Zi330、TS6278、Zheng28、832、JiA-034、Shen5003、HuangC、Si287、4866
HAP10	23	Si533、Ji992、C416、Mo17、Dan1324、Ji477、Jan-67、7922、K22、Shuang105、Ji046、5022(B)、Jinhuang76、Shen3336、He344、K12、B104、Danhuang02、Huang428-3、5Gong、416、53Xuan3、Dong156
HAP11	1	Jan-37
HAP12	8	C649、502、LX9801、Liao540、Liao184、Dan360、Dan340、Ji81162
HAP13	1	2002F22
HAP14	1	CA339
HAP15	1	Si273
HAP16	1	Zhonghuang204 R2040

Table S7 changes of amino acids corresponding to SNP of *ZmMYBR24* gene

Number	SNP sites	Changes in amino acids	basic mutation
SNP79	79	A/T	GCG/ACG
SNP153	153	S/S	TCG/TCA
SNP201	201	E/D	GAG/GAC
SNP211	211	P/S	CCC/TCC
SNP240	240	A/A	GCC/GCA
SNP259	259	A/T	GCC/ACC
SNP290	290	P/L	CCG/CTG
SNP300	300	S/S	TCG/TCC
SNP345	345	A/A	GCA/GCT
SNP417	417	K/N	AAG/AAT
SNP480	480	G/G	GGC/GGG
SNP493	493	R/R	AGA/CGA
SNP621	621	A/A	GCA/GCT
SNP694	694	A/P	GCC/CCC
SNP706	706	A/P	GCA/CCA
SNP788	788	A/V	GCC/GTC
SNP803	803	N/S	AAC/AGC
SNP814	814	L/M	CTG/ATG
SNP954	954	S/S	AGT/AGC
SNP969	969	P/P	CCG/CCA
SNP1002	1002	Q/Q	CAG/CAA
SNP1019	1019	Q/P	CAG/CCG
SNP1041	1041	T/T	ACG/ACA
SNP1047	1047	P/P	CCC/CCA

SNP1051	1051	L/M	CTG/ATG

Table S8 80 inbred lines in this study

Code	Inbred lines	Code	Inbred lines
1	SH15	41	Zhongzong4C1-3-2-2-b-5-2-1-b
2	Chuan273	42	Zong31
3	Y7	43	CA339
4	Jinhuang55	44	Ji63
5	Shen3336	45	888-9
6	Ji412	46	Chong72
7	Ji465	47	Si273
8	77	48	Liao540
9	Guan17	49	Liao184
10	706Fu	50	Suixi707
11	Si533	51	Dan360
12	Ji992	52	Dan340
13	C416	53	DH34
14	JiA-034	54	B84
15	Mo17	55	Ji81162
16	Dan1324	56	7884
17	Ji846	57	5022(B)
18	Ji477	58	Si387
19	He344	59	6523
20	C649	60	B104
21	J001	61	2002F22

22	D387	62	32
23	K12	63	832
24	502	64	8129
25	LX9801	65	TS6278
26	Si-279	66	Jinhuang63
27	CAL70	67	Jinhuang73
28	24473	68	Jinhuang76
29	13516	69	Qing795
30	1923004	70	Shuang105
31	Ji046	71	Zhonghuang204 R2040
32	Shen5003	72	Danhuang02
33	7922	73	Huang428-3
34	803	74	HuangC
35	K22	75	5Gong
36	K10	76	Si287
37	Dong46	77	4866
38	Zi330	78	416
39	Chang3	79	53Xuan3
40	Zheng28	80	Dong156

Table S9 The primers used in this study

Experiment	Name	Sequences（5'→3'）	CG（%）	Amplification length（bp）
Mutant strain detection	MYB-F2-574	GCTCCGTTTCCGTTCA	-	-
	MYB-R2-574	GCAGGTCATTCCCGACT	-	-
Quantitative fluorescence in the analysis of gene expression patterns	Q*ZmMYBR24*-F	CATCTCCCGGCACTTCG	-	-
	Q*ZmMYBR24*-R	TGGACCTGCGCTTCTTGT	-	-
	Actin-F	GTTGGGCGTCCTCGTCA	-	-
	Actin-R	TGGGTCATCTTCTCCCTGTT	-	-
qRT-PCR of DEGs	*Zm00001d046235*-F	TGTAGCATACATACTACTCGTGC	-	-
	Zm00001d046235-R	CCGCATAACTAATTTGGCTGAG	-	-
	Zm00001d013654-F	GCTCGTCTATTACCCGCTCG	-	-
	Zm00001d013654-R	ACGAACCAGATCTTGAGCCC	-	-
	Zm00001d042446-F	CTTCCCAGTGACAGGCTGAT	-	-
	Zm00001d042446-R	CGAGACTGGCACAATCAAACC	-	-
	Zm00001d004248-F	TCCTGTCGCTGCTATTG	-	-
	Zm00001d004248-R	ATGCTTCGTCCCTCTGT	-	-
	Zm00001d021168-F	CGTCGGCTGCTTCTTCA	-	-
	Zm00001d021168-R	CGTTGGTCGTCTGGTCC	-	-
	Zm00001d053938-F	GGACTGGGTGAAGCG	-	-
	Zm00001d053938-R	GGCAGGGTGGGGTAG	-	-
	Zm00001d011649-F	CGTGCCCGCCTACTT	-	-
	Zm00001d011649-R	TCCTTCCCCATGTCC	-	-
	Zm00001d022475-F	GATGGCGACCCCTAC	-	-
	Zm00001d022475-R	CCAGCACCAAGCACA	-	-
Homologous recombination	BK-EcoRI-MYBR24	CTGCATATGGCCATGGAGGCCGAATTCATGGCTAGGAAGTGCTCTAGC	-	-

	BK-BamHI-MYBR24	TGTGGGAA<br>ATGCGGCCGCTGCAGGTCGACGGATCCTTACGTGACCCTGATGGTGCC<br>CAGAAA	-	-
Primer cloning of *ZmMYBR24* gene sequence	MYB-1F	CATCCTCCCTCTTCTCCTG	57.9	1146
	MYB-1R	GCGATTATCGCATCCG	56.3	
	MYB-2F	AGCCACGCCCAGAAGTA	58.8	615
	MYB-2R	CGTGATAAACGACCGCAT	50.0	

Table S10 Methods for evaluation of target traits

Trait	Abbreviation	Unit	Method of testing phenotype
Germination percentage	GR	-	The number of seeds with radicle lengths exceeding 0.5 cm was counted on day 7.
Seeding Length	SL	cm	The aboveground portion of maize seedlings is defined as the region extending up to the highest point where leaf expansion occurs.
Shoot Fresh Weight	SFW	g	The fresh weight of the plants was measured after they were cut at the base.
Root Fresh Weight	RFW	g	The roots were rinsed with distilled water, blotted dry using filter paper, and subsequently measured for fresh weight.
Shoot Dry Weight	SDW	g	The aboveground fresh samples were subjected to deactivation at 105°C for 30 minutes, followed by drying at 80°C for 24 hours. After reaching a constant weight, measurements were taken.
Shoot Dry Weight	RDW	g	The root fresh samples were subjected to deactivation at 105°C for 30 minutes, followed by drying at 80°C for 24 hours. After reaching a constant weight, the measurements were conducted.
Root Length	RL	cm	The scanning process was performed using an Epson Perfection V800 scanner, and the data analysis was conducted using the Regent WinRhizo Canada software.
Root Volume	RV	Mm	
